# Silent practices becoming norms: planned napping for nurses during intensive care night shifts – a focus group study

**DOI:** 10.1177/17449871251401036

**Published:** 2026-02-21

**Authors:** Carita Löfqvist, Johanna Kaarina Siivonen, Anna Axelin, Laura-Maria Peltonen, Marita Ritmala

**Affiliations:** Doctoral Researcher, Department of Nursing Science, University of Turku, Turku, Finland; Master of Health Sciences, Department of Nursing Science, University of Turku, Turku, Finland; Professor, Department of Nursing Science, University of Turku, Turku, Finland; Associate Professor, Department of Health and Social Management and Kuopio University Hospital, University of Eastern Finland, Finland; Magnet Program Director, Nursing Administration, Helsinki University Hospital, Finland

**Keywords:** fatigue prevention, health policy, intensive care units, shift work schedule, sleep, planned napping, qualitative research

## Abstract

**Background:**

Night shifts in intensive care units (ICUs) are associated with significant physical and cognitive fatigue among nurses, which may affect staff well-being and patient safety. Although short naps have been shown to reduce fatigue, their implementation in ICUs remains limited and poorly understood.

**Aim:**

This study explored ICU nurses’ and nurse managers’ perceptions of planned napping.

**Methods:**

A qualitative descriptive design was used, involving nine focus group interviews (*n* = 20) across three Finnish ICUs. Data were analysed using inductive content analysis.

**Results:**

Participants described severe fatigue during night shifts and acknowledged the benefits of planned napping, including improved alertness, reduced errors, and enhanced well-being. Informal napping was common, but formal structures were lacking. Barriers included unclear policies, cultural resistance, and logistical challenges; whereas facilitators involved organisational culture, environment, scheduling, and managerial support. Emphasis was placed on fairness, flexibility, and clear protocols.

**Conclusions:**

Planned napping was perceived as a valuable strategy for managing fatigue, but successful implementation requires institutional support and context-sensitive planning. This study provides practical insights into implementing planned napping in high-acuity environments and supports the development of structured, evidence-informed protocols to promote staff well-being, patient safety and sustainable nursing practices.

## Introduction

Night shifts are demanding for nurses, requiring constant alertness and the ability to provide high-quality care and ensure patient safety (Imes et al., 2023). Intensive care nurses must master a broad range of clinical skills, including the assessment of respiratory, circulatory, and neurological status (Higginson and Jones, 2009). They must also operate complex equipment such as ventilators (Vatani et al., 2021) and make rapid, accurate clinical decisions (Kolagari et al., 2023). Night shifts have significant disadvantages for nurses’ and patients’ health. Fatigue from night shifts negatively impacts patient care quality and safety (Di Muzio et al., 2019). Studies have shown that for nurses, night shifts disrupt the natural sleep–wake rhythm, leading to sleep deprivation and significant fatigue (Dutheil et al., 2020; Querstret et al., 2020; Sallinen and Kecklund, 2010; Weaver et al., 2024). Intensive care nurses in particular can face stress, chronic fatigue, and work-related sleep disorders due to rotating shifts (Imes and Chasens, 2019; Membrive-Jiménez et al., 2022).

Given the challenging implications of night shifts for both nurses and patient care, it is essential to explore strategies that support staff well-being and maintain patient safety. Naps are referred to as short periods of sleep, often consisting of light sleep, that can refresh the mind, improve memory, and support learning (Mednick et al., 2003). Studies show that providing rest areas and allowing short, planned naps during night shifts can reduce shift work sleep disorder symptoms (Yu et al., 2025). A brief planned nap during a night shift offers several benefits to nurses. It can effectively combat fatigue (Geiger-Brown et al., 2016), improve well-being (Fallis et al., 2011; Smith-Coggings et al., 2006; Zion and Schochat, 2019), reduce stress (Oriyama et al., 2014) and decrease the risk of drowsy driving (Geiger-Brown et al., 2021). Planned napping also aids recovery after work and has positive physiological effects (Palermo et al., 2015; Oriyama et al., 2014). International guidelines recommend short, planned naps during work shifts to reduce fatigue and enhance alertness. For example, The Joint Commission (2011) and the National Institute for Occupational Safety and Health (Centers for Disease Control and Prevention, National Institute for Occupational Safety and Health, n.d.), advocate scheduled napping as an effective fatigue countermeasure.

Despite these benefits, planned napping during night shifts is not widely implemented (Li et al., 2019). Barriers include lack of organisational support (Fallis et al., 2011; Ruggiero & Redeker, 2014; Scott et al., 2010; Trinkoff et al., 2021) and a general disapproval of planned napping during night shifts (Landis et al., 2021). For successful implementation, planned napping routines should be clearly defined and structured (Gamble and Foran, 2021; Ruggiero and Redeker, 2014), and a protocol is needed (Alnawafleh, 2024). Although planned napping interventions have been examined in general hospitals, emergency departments and acute care settings (Geiger-Brown et al., 2021; Smith-Coggins et al., 2006; Zion and Shochat, 2019), their application in intensive care units (ICUs) remains unexplored. Moreover, the factors that influence the successful implementation of planned napping strategies in such high-acuity environments are not yet well understood. Unlike in other units, ICU nurses typically bear individual responsibility for highly unstable patients, which complicates both the logistics and professional acceptability of planned napping. Given the complexity of intensive care settings and the demanding nature of ICU nursing, it is essential to generate context-specific research evidence to inform practice.

### Aim

This study aims to describe the perceptions of nurse managers and registered nurses regarding planned napping during night shifts in ICUs. The goal is to provide knowledge of what should be considered when implementing planned napping for nurses on night shifts in the ICU.

## Methodology

### Study design

A qualitative descriptive design (Holloway and Galvin, 2024) was used, and data were analysed using thematic analysis (Braun and Clarke, 2006) from a constructionist perspective, focusing on how participants’ accounts reflected on planned napping during night shift work. Standards for Reporting Qualitative Research (SRQR) guidelines were used to ensure transparent reporting (O’Brien et al., 2014).

### Participants and data collection

Data were collected through nine qualitative focus group interviews conducted between March 2022 and January 2023 in three general ICUs in Finland – two in university hospitals and one in a municipal hospital.

Participants were recruited using purposive sampling, ensuring a broad and comprehensive understanding and expertise of ICU care (Polit and Beck, 2018: 164). Each unit contributed at least two nurse managers or assistant nurse managers and two registered nurses working rotating shifts. Eligibility criteria included registered nurses currently employed in ICU and engaged in shift work. Recruitment was facilitated via email by each unit’s nurse manager. In total, 20 participants (aged 29–61, 16 female) with 4–30 years of ICU experience were included.

Due to the sensitivity of the topic, nurse managers and registered nurses were interviewed in separate groups. Despite the aim to facilitate dynamic group interaction, most focus groups consisted of only two participants, with the largest group including four. The size of each interview group was determined by the availability of participants who were both scheduled to work and willing to take part on the respective interview days. Focus group characteristics are summarised in Table 1. Interviews were guided by a framework (Table 2) developed from literature and predefined research objectives. Themes included: night shift responsibilities, attitudes towards planned napping, nap duration and timing, napping environment, evidence-based knowledge on napping, and considerations for implementation.

**Table 1.** Focus group characteristics.

Focus group	Number of participants	Role
1	2	Managerial
2	2	Nursing
3	2	Nursing
4	2	Nursing
5	2	Managerial
6	2	Nursing
7	2	Nursing
8	2	Managerial
9	4	Nursing
**Total**	**20**	

**Table 2.** Interview guide.

Themes	Examples of questions to support the discussion
Night shift responsibilities	What kind of agreed-upon practices do you currently have during breaks at night shifts? Do you have allocated rounds or other agreed work tasks during the night shift?
Attitudes towards planned napping	What thoughts do you have about planned napping during night shift? What aspects can promote or hinder this practice (planned napping)
Nap duration and timing	What would be the ideal length of a planned nap during night shift? Is there something to consider? Can or should the length of a planned nap be verified? What should be considered at the timing of the nap?
Napping environment	What kind of space should be for a planned nap? What should be considered for the surroundings? Are there any supplies that would be needed?
Evidence-based knowledge on planned napping	What kind of information would be needed if planned napping was be implemented?
Considerations for implementation	To make planned napping for the night shift feasible, what other aspects should be considered when planning the implementation?

All focus groups were conducted on-site by the first author, who had not previously interacted with the participants. The duration of the focus groups ranged from 37 to 80 minutes, with a mean length of 60 minutes. Data collection continued until thematic saturation was reached within each focus group, and no new themes emerged across subsequent groups, indicating overall saturation. The participants shared their experiences openly, extensively and in detail. All sessions were audio-recorded, pseudonymised, and transcribed verbatim. Participant characteristics are summarised in Table 3.

**Table 3.** Participant characteristics.

Characteristics	*n* (%)
Gender	
Female	16 (80)
Male	4 (20)
Nurse manager or assistant nurse manager	6 (30)
Registered nurse	14 (70)
	Mean
Age	45
Years of professional experience in ICU (nurse or manager)	
Nurse manager or assistant nurse manager	6
Registered nurse	12

### Setting

This study was conducted in mixed-care ICUs, where both medical and surgical patients were treated. These units provide specialised care for critically ill patients, including those with multiple organ failure, severe neurological conditions and major surgical needs. According to brief unit descriptions provided by nurse managers and assistant nurse managers, the number of patient beds per unit ranged from 9 to 26. All units operated with a Medical Emergency Team (MET) and employed from several dozen to nearly 200 nurses.

Nurses worked in teams, with team size flexibly adjusted based on patient acuity and required clinical expertise. During night shifts, nurses typically cared for one or two patients. Each shift began with a team briefing where the work assignment was finalised by the charge nurse according to the patient acuity situation.

All nurses worked night shifts unless medically exempt. Although some nurses worked night shifts occasionally, others did so regularly or exclusively. Shifts lasted a minimum of 10 hours and 45 minutes, with an average duration of 12 hours, typically starting between 7:00 and 9:00 p.m. and ending between 7:00 and 8:00 a.m. Night shifts were described as mentally demanding and variable. Nurses aimed to minimise non-essential activity during these hours, focusing on essential care and procedures.

Breaks during night shifts were typically taken two to three times per shift, without fixed schedules and were adapted to patient needs. Nurses remained reachable during breaks, often carrying phones, though interruptions were infrequent. Break coordination was managed within teams, with some preferring structured routines and others opting for flexibility. MET nurses and charge nurses could assist in facilitating breaks, which were usually taken in break rooms or at the nurses’ station.

### Data analysis

The data were analysed using inductive content analysis (Elo and Kyngäs, 2008), following the steps of familiarisation, open coding, categorisation and abstraction. The data were independently analysed by two researchers (CL and JS), after which the results were compared and collaboratively refined (CL and JS). To gain an overall understanding and identify preliminary themes, interview transcripts were first summarised. The data were subsequently imported into NVIVO (Version 14; QSR International Pty Ltd, 2022), where meaning units were coded and grouped into subcategories based on thematic similarity. Participant validation was not conducted. Instead, credibility was supported through peer debriefing and careful documentation of the analytic process.

Codes were organised in Microsoft Excel (Version 2408; Microsoft Corporation, 2024) to facilitate comparison and refinement. Subcategories were named and further abstracted into main categories. The process was iterative and guided by the research question to ensure a comprehensive and detailed analysis of participants’ perceptions (Graneheim and Lundman, 2004).

### Ethical considerations

The study followed accepted scientific practices (Finnish Advisory Board on Research Integrity TENK, 2023). Organisational permissions were obtained prior to data collection. According to national legislation, ethical review was not required, as the study did not constitute medical research or involve physical or psychological interventions (Finnish National Board on Research Integrity TENK, 2023). All participants provided informed consent and were informed of their right to withdraw at any time without providing a reason. No reimbursement was offered for participation. Participants were pseudonymised upon entry into the data management system to ensure confidentiality.

## Results

**Figure 1.**
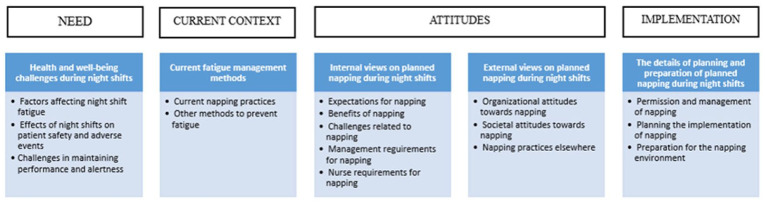
Overview of the main findings related to the perspectives of planned napping in night shifts in ICU environment.

The analysis of the interview data revealed four overarching thematic domains that reflected participants’ perspectives on planned napping during night shifts in the ICU: Need, Current Context, Attitudes and Implementation (Figure 1). These domains encompass the main categories and sub-categories derived from the data. Participants described various health and well-being challenges associated with night work, including fatigue-related factors and concerns about patient safety, which highlighted the perceived need for effective fatigue mitigation strategies. Their current approaches to managing fatigue included different methods, such as existing informal napping practices and other alertness-promoting strategies, offering insight into the practical context in which structured interventions might be introduced.

Attitudes towards planned napping emerged as a central theme, encompassing both internal and external viewpoints. Participants discussed expectations, perceived benefits, and challenges related to planned napping, as well as the requirements from both management and nursing staff. External perspectives included organisational culture, societal norms and references to practices in other healthcare settings. Finally, participants shared detailed considerations for the practical implementation of planned napping during night shifts, including the need for managerial permission, structured planning and preparation of the physical environment to support effective rest.

### Health and well-being challenges during night shifts

The managers viewed the harmful effects of night work broadly, whereas the nurses described them through personal and peer experiences. Morning chronotype nurses were described as having had greater difficulty in both preparing for and performing night shifts. The nurses reported difficulties in sleeping before or between shifts, especially during consecutive night shifts. Regular night shift workers experienced less fatigue than those with irregular schedules, though family responsibilities still posed challenges.

The first night shift was often described as the hardest, with fatigue peaking between 2 and 5 a.m. Nurses were aware of health risks and expressed concern about exhaustion and absenteeism. They described night shifts as surviving moment by moment, with extreme stress and physical discomfort. The feeling of discomfort was even described as a feeling of almost dying.

I feel so physically bad that I honestly sometimes think I might die. Like I’m having a heart attack or something. (Nurse ID2)

Fatigue was intensified by efforts to maintain a calm, sleep-supportive environment for patients, with dimmed lights and reduced noise. Nurses’ fatigue was also influenced by patient condition: unstable patients kept nurses alert, whereas stable ones increased drowsiness. Isolation rooms were particularly challenging due to darkness, silence, and solitude. The constant sound of ventilators and a possible use of face masks further increased fatigue, and some nurses reported dozing off due to lack of stimulation.

Despite fatigue, nurses felt a strong responsibility to remain alert. They believed the risk of medication errors and adverse events was highest in the early morning. One unit had discontinued morning medication rounds due to safety concerns. Nurses admitted to unintentionally falling asleep at the bedside. Although seen as human, such incidents were viewed as endangering patient safety. According to the nurses, fatigue impaired attention, alertness, and memory and led to mistakes and poor end-of-shift reporting.

Yeah, in the early morning hours you really start to worry–like, I hope no acute patient shows up all of a sudden. Of course, then you get that rush of adrenaline and you can function after a bit. But your head’s all foggy. (Nurse ID1)

You (nurses) really do have a responsibility for another human being. Even when you’re tired, you have to stop and ask yourself – am I actually fit to work or not? You have to remember, you’re responsible for keeping that patient alive during their care. (Nurse Manager ID6)

Difficulties were also encountered after night shifts including slow recovery, with effects felt the next day. Recovery was even slower after busy and stressful nights. Family life and hobbies suffered due to post-shift fatigue and disrupted sleep–wake rhythms, leading to missed exercise and sports. The participants reported drowsy driving after a night shift, which posed a significant safety risk, with some nurses reporting episodes of falling asleep at the wheel.

### Current fatigue management methods

Both managers and nurses acknowledged the occurrence of napping during night shifts, despite the absence of a formal policy on the practice. Managers encouraged napping when signs of fatigue were evident. Even when managers expressed a permissive stance towards planned napping, nurses reported uncertainty regarding its acceptability, which led to hesitation or avoidance.

But the fact that you’d be allowed to do it–that would be a good thing, at least from my point of view. Because it always brings up the issue that there are people who sleep anyway, while others try to just push through. (Nurse ID1)

Unit-level practices varied considerably. According to the nurses, naps were typically taken during breaks or arranged through an informal rotation system among colleagues. Alternatively, nurses reported informing coworkers prior to resting and employing precautionary measures – such as setting monitor alarms louder – to ensure they would awaken to the alarm.

So yeah, I’d say the most effective thing (reflecting on her previous role as a bedside nurse) is having my own chair right next to the patient. I kind of doze off a bit there, you know, just resting. But I can snap out of it if I hear any beeping or something like that. (Nurse Manager ID5)

Nurses described employing a variety of strategies to maintain alertness during night shifts. Environmental adjustments, such as keeping the lights on or engaging in brief conversations with colleagues, were used to counteract fatigue. Nurses preferred to stay at the patient’s bedside, occupying themselves with low-effort activities – such as browsing the computer or knitting – while simultaneously monitoring the patient’s condition. Short walking breaks were also reported as a method to prevent fatigue. In addition to physical activity, the nurses reported using break periods for relaxation rather than for eating, highlighting the diverse functions of rest during night shift.

### Internal views on planned napping during night shifts

Participants generally expressed positive attitudes towards planned napping during night shifts. Planned napping was perceived as reducing stress and enhancing preparedness for night work. Although the managers were generally supportive of the practice, their views on the necessity of formal policy varied: some emphasised the importance of clear guidelines to ensure equality and legitimacy, whereas others considered formalisation unnecessary, advocating instead for flexible, team-level implementation. In contrast, the nurses expressed a unanimous view that clear guidelines were essential to ensure fairness and to foster openness regarding fatigue-related concerns. Both the nurses and managers emphasised the importance of organisational-level endorsement – such as approval from senior management or the head nurse – to ensure consistency and signal managerial understanding and support for the unique demands of night work, and to ensure legitimacy and consistency across units.

The issue was also considered from the perspective of roster planning, with the participants noting that formalising planned napping practices could increase nurses’ willingness to work night shifts by making them less burdensome. Moreover, the nurses believed that institutionalising planned napping would likely be necessary across the healthcare sector, drawing parallels to its established acceptance among physicians. Participants highlighted that leadership endorsement could normalise planned napping practices and reduce hesitation among staff. They noted that the decision should reflect a basic-level commitment to supporting nurses. However, not all participants were optimistic about the likelihood of receiving such permission. Although most participants supported the idea of shared policies, concerns were raised regarding the potential for disagreements if rules were inconsistently applied or perceived as unfair.

And I personally feel it’d be really good if this became a common practice that it’s okay to take a nap/sleep with permission, because right now it’s kind of vague—some of the more experienced folks look down on you a bit if you nap. (Nurse ID8)

Both the nurse managers and nurses considered planned napping as beneficial for physical health, well-being, job satisfaction and patient safety. The participants believed that planned napping reduced clinical errors, improved alertness and supported post-shift recovery. Furthermore, both the nurses and managers noted that planned napping could improve attitudes towards night shifts and even extend careers. Comparisons were made to other industries, such as manufacturing, where planned napping practices have been associated with increased efficiency, suggesting that similar benefits could be realised in healthcare.

I think it would definitely improve patient safety—those early morning hours when everything’s kind of spinning and you can barely remember the difference between Furosemide and Norepi. (Nursing manger ID2)

In addition to the benefits of planned napping, the managers and nurses identified several challenges in its implementation. Resource constraints were described as a major barrier, particularly in smaller units or in situations involving staff absences. Organising long naps was also considered difficult due to high workload demands and the need to always ensure adequate clinical competence on the unit. These were found to limit the flexibility required to implement planned napping practices consistently across different settings.

Even in larger units, identifying appropriate spaces for planned napping was reported to be challenging. Despite spatial constraints, the nurses noted that absolute equality in nap access was not essential, as equitable access was believed to even out in the long term. Specific challenges were noted for nurses in specialised roles, such as MET nurses, who were often considered unable to rest due to the lack of available backup staff. Nurses also described personal barriers to planned napping, including difficulty in relaxing during shifts or concerns about post-nap grogginess.

I’m in such a daze that I’d just keep sleeping. I wouldn’t wake up properly. That makes me wonder about this (napping). (Nurse ID14)

Implementation of planned napping practices was seen to require staff flexibility and situational awareness. Participants emphasised that planned naps needed to remain adaptable and could be postponed or cancelled at short notice to ensure continuity of care. The ability to negotiate planned napping time based on real-time clinical demands was considered essential for maintaining workflow and team cooperation.

If it’s like, a certain time comes and you’re busy with a patient, then you just have to say: ‘Right now I can’t go. Would it be okay if I have my nap once things have calmed down a bit—or something like that.’ Like could you go then? (Nurse Manager ID4)

Accessibility during resting periods was also highlighted. Most felt that nurses should remain reachable, for example, by keeping the bedside telephone accessible in case of sudden changes in a patient’s condition. In some cases, informing colleagues of one’s location was seen as a sufficient way to ensure accessibility.

### External views on planned napping in night shifts

Concerns were expressed regarding how planned napping might be perceived by upper management. The participants noted that the ability to rest during night shifts could be misinterpreted as a sign of a reduced workload or operational inefficiency, potentially leading to staffing reductions. The perception that planned napping equates to inactivity was seen as a risk in justifying the current level of night shift staffing.

There were also concerns about equity across hospital units. The implementation of planned napping practices exclusively in intensive care settings was thought to potentially generate tension among staff in other departments. The participants emphasised that if planned napping were to be accepted, it should be made available across the organisation.

The nurses reflected on broader societal and cultural attitudes that shaped perceptions of planned napping during night shifts. The hospital environment was described as conservative, characterised by a strong work ethic and a professional identity rooted in self-sacrifice and moral responsibility. Within this context, nursing was often viewed as a calling, where resting – particularly planned napping – was historically condemned and perceived by many as inappropriate, even during designated breaks.

Yeah, that really marks this field—the idea of what kind of nurse is seen as good or how a nurse is supposed to do a good job. It’s always that hardworking type, the one who’s constantly running around, coming and going. (Nurse ID3)

But I’ve got to say, the hospital world is still pretty old-fashioned here in Finland—like, there’s still very much this idea that sleeping just isn’t something you do. (Nurse manager ID2)

The nurses also discussed the impact of increasing efficiency demands in healthcare. They emphasised the need to broaden the understanding of efficiency to include practices that support well-being and sustainable work. Recognising the legitimacy of planned napping in relational and people-centred professions was seen as an important step towards changing attitudes. Although some nurses observed a gradual shift towards greater empathy for nurses’ fatigue, others noted that broader cultural change would be necessary to gain full cultural acceptance for planned napping.

The participants questioned the absence of formal policies regarding planned napping within Finnish healthcare. Awareness of planned napping practices in other units, such as operating rooms, and in international contexts prompted reflections on why similar guidelines had not been adopted locally and for nurses. Comparisons were drawn to fields such as aviation and manufacturing, where structured naps were described as standard and integral to safety and performance. In aviation, for example, resting when fatigued was not only accepted but encouraged, reinforcing the idea that planned napping can be a professional responsibility rather than a sign of inefficiency.

### The details of planning and preparation of planned napping during night shifts

Obtaining formal permission was regarded as essential. Once approval was granted, the participants believed that clear rules could be developed regarding planned napping duration, conditions for interruption and responsibilities during napping periods. The nurses expressed a desire to be involved in the planning process, and this was also supported by the managers. The participants emphasised the importance of evidence-informed decisions. Transparent communication through official channels was considered essential to ensure the shared understanding and acceptance of the practice.

There would be this own space, a quiet space just for sleeping, so you wouldn’t go there to do anything else really. And then those who don’t want to use it for sleeping could use it just for resting. (Nurse ID5)

Organising planned napping at the shift and team level was considered a practical approach. The participants suggested aligning naps with existing meal breaks. It was also proposed that the nurse in charge could assist in coordinating nap times during busy periods, although it was acknowledged that this role is often already heavily burdened with other responsibilities. Participants expressed confidence in the ability of teams to self-organise rest periods in a way that supports both individual recovery and unit functionality. Reported views on appropriate nap duration ranged from 15 minutes to 2 hours, with shorter naps of 15–30 minutes generally considered more feasible within the constraints of clinical work. Suggested timing for naps fell within the early morning hours between 2 and 6 a.m., though strict scheduling was considered impractical due to the dynamic nature of patient care.

Preferences for the planned napping environment emphasised the importance of privacy, quietness, and darkness. The participants identified several potential spaces for rest, including unused isolation rooms, break rooms, and library areas. The availability of appropriate furnishing and equipment – such as beds, linens, shelves for personal items, and charging outlets – was considered important for creating a restful setting. The nurses noted that, if desired, items such as earplugs or sleep masks could be brought from home to enhance comfort and reduce disturbances during rest.

We do have a few extra beds, so if you want to lie down for a bit, you can. There’s no dedicated space for it or anything, but you can usually manage to get that half-hour break. (Nurse manager ID2)

## Discussion

This study explored how ICU nurses and nurse managers perceive planned napping during night shifts, and why it should be implemented in the intensive care environment. By focusing specifically on the intensive care context, an area not extensively examined in previous research, this study offers new insights. The findings indicate that implementing a fatigue management strategy such as planned napping during night shift is a complex intervention that requires careful consideration of multiple interrelated factors. The demanding nature of the ICU environment, combined with the need for sustained vigilance among nurses, further complicates the adoption of such practices.

Participants clearly emphasised the need for effective fatigue management strategies, identifying fatigue as a significant and often severe challenge during night shifts. Their accounts highlighted not only the intensity of night shift fatigue but also echoed earlier findings (Weaver et al., 2024). Both nurses and nurse managers acknowledged the negative consequences of fatigue, discussing the issue with shared understanding and empathy. Difficulties related to recovery and maintaining work–life balance reflected themes reported in previous literature (Trinkoff et al., 2021).

This study contributes to existing literature by showing that sleeping during night shifts occurs in practice, primarily as informal or unspoken behaviour. Although participants described various fatigue management strategies, such as activation techniques and environmental adjustments, planned napping emerged as a central theme. The findings underscore that fatigue management should be addressed as an organisational responsibility rather than left to individual coping mechanisms. Although both intentional and unintentional napping were recognised as part of night shift reality, it lacked formal support. This ambiguity led to inconsistent and unequally applied practices across units, potentially leading to silent practices, unequal working conditions, and increased risk of fatigue-related errors. Both nurses and nurse managers emphasised the need for clear, organisation-wide guidance, preferably mandated by higher-level management rather than developed independently at the unit level.

Attitudes towards planned napping varied: some nurses viewed it as acceptable, whereas others expressed uncertainty or concern about its legitimacy. These mixed views align with earlier studies (Dalky et al., 2018; Fallis et al., 2011), and participants voiced a strong desire for equitable and clearly defined rest opportunities. At the policy level, the results of this study highlight the need for national guidelines and institutional frameworks that recognise rest and recovery as essential to safe and sustainable healthcare work. Participants questioned the absence of planned napping policies in Finnish healthcare, noting that similar guidelines exist in other units and countries. Despite existing global recommendations, such as those from the Joint Commission (2011) and the National Institute for Occupational Safety and Health (Centers for Disease Control and Prevention, National Institute for Occupational Safety and Health, n.d.), which advocate short, planned naps to reduce fatigue and enhance alertness, these practices remain underutilised. This points to a persistent gap between evidence-based guidelines and their implementation in clinical settings.

The absence of written policies revealed strong internal and external attitudes towards planned napping. Internally, participants not only raised practical concerns, such as the feasibility of sleeping in the ICU environment, but also expressed a broader desire for cultural change within healthcare. Many hoped for alignment with other safety-critical fields, where institutional interventions actively support staff alertness and functional capacity. Externally, attitudes were shaped by the perception of nursing as a calling, in which napping is often stigmatised. Previous research has linked this vocational view to self-sacrifice and passivity, potentially limiting nurses’ willingness to advocate for improved working conditions (Kallio et al., 2022). These underlying beliefs may act as barriers to implementing planned napping and should be addressed proactively. Organisational support is needed at all levels, from bedside nurses to administration, in order to enable both structural and cultural change. Utilising change management practices such as vision creation, effective communication, employee involvement, and supportive leadership have been shown to significantly enhance employee commitment to change (Onyeneke and Abe, 2021).

Finally, the importance of careful implementation was strongly emphasised. Written protocols and the active involvement of nursing staff in the planning process were seen as essential, aligning with best practices identified in earlier studies (Geiger-Brown et al., 2021). These findings reinforce the need for implementation strategies that are sensitive to unit-specific cultures and norms, as previously recommended (Geiger-Brown et al., 2016). Given the sensitive and complex nature of intensive care nursing, it is crucial to address the contextual factors that influence both the planning and execution of interventions such as planned napping during night shifts. To support the development of evidence-based practices, future research should include also policy pilot studies and organisational trials, which could provide valuable insights into the feasibility and impact of planned napping interventions in healthcare settings.

### Strengths and limitations of the study

This study applied the credibility, dependability, and transferability in line with the trustworthiness of qualitative content analysis (Elo and Kyngäs, 2008; Graneheim and Lundman, 2004). Credibility was supported by interviews that enabled participants to describe and justify their experiences in depth. This approach allowed for a nuanced understanding of the phenomenon and ensured that the findings were grounded in the participants’ own perspectives. Dependability was enhanced through a systematic and transparent thematic analysis process. Transferability was supported by the inclusion of registered nurses and nurse managers from three different ICUs across Finland. The diversity of perspectives provided a rich dataset and increased the relevance of the findings beyond the immediate study context.

The main limitation of this study concerns the composition of the focus group interviews. Although the recommended group size is typically 5 to 12 participants to encourage dynamic interaction (Gray and Grove, 2021), most groups in this study consisted of only two participants, with the largest group including four. This may have limited the depth of group discussion. Nevertheless, the conversations were rich and varied, and data saturation was achieved, lending robustness to the findings. In addition, there is a possibility of self-selection bias, as participants willing to take part may have been more open or positively inclined towards discussing planned napping practices. Although the study was conducted in ICU settings, the identified themes – including fatigue, inconsistent napping practices and organisational policies – are likely relevant across various healthcare environments. A detailed contextual description enables assessment of the applicability of the findings across different settings, thereby supporting the transferability of the results.

## Conclusion

To sustain night shift work in intensive care and uphold the vigilance requires in high-acuity environments, targeted strategies to support nurse endurance are essential. This study emphasises the need for context-specific fatigue prevention measures, such as planned napping, supported by evidence-based recommendations. The ICU’s complexity and safety-critical nature demand careful consideration in implementing such interventions. Findings reveal a readiness for change among nurses and managers, yet formal guidelines and persistent stigma around napping remain barriers. Embedding planned napping opportunities into organisational policy could be a strategic move to enhance workforce sustainability and the long-term attractiveness of the nursing profession.

### Implications for future research

Given the identified challenges related to fatigue, inconsistent practices and lack of formal support, future research should focus on the design and testing of planned napping interventions specifically tailored to the ICU context, considering its unique demands, safety requirements, and organisational culture. In addition to assessing the effectiveness of planned napping, attention should also be given to the implementation process. The Promoting Action on Research Implementation in Health Services (PARIHS) framework (Rycroft-Malone, 2004), which emphasises the interaction between evidence, context and facilitation, could provide a relevant foundation for examining implementation.

**Key points for policy, practice and/or research**

- Fatigue among ICU nurses has been identified as a profound and multifaceted issue, impacting clinical performance, patient safety, personal well-being, and work–life balance.
- The complexity of the ICU environment – including its intensity, safety demands and cultural norms – must be considered when implementing planned napping interventions.
- Both nurses and nurse managers expressed a clear need, willingness, and openness towards planned napping as a fatigue management strategy, suggesting favourable conditions for implementation.
- Prevailing cultural attitudes and the absence of formal policies have been recognised as significant barriers to implementation and should be addressed through clear institutional guidelines and organisational support.
